# Genome Skimming Reveals Plastome Conservation, Phylogenetic Structure, and Novel Molecular Markers in Valuable Orchid *Changnienia amoena*

**DOI:** 10.3390/genes16070723

**Published:** 2025-06-20

**Authors:** Rui-Sen Lu, Ke Hu, Yu Liu, Xiao-Qin Sun, Xing-Jian Liu

**Affiliations:** Jiangsu Key Laboratory for Conservation and Utilization of Plant Resources, Institute of Botany, Jiangsu Province and Chinese Academy of Sciences, Nanjing 210014, China; lurs@cnbg.net (R.-S.L.); rabbitophelia@163.com (K.H.); clifford.y.liu@outlook.com (Y.L.); xiaoqinsun@cnbg.net (X.-Q.S.)

**Keywords:** *Changnienia amoena*, plastome, comparative genomics, phylogenetic analysis, molecular markers

## Abstract

**Background/Objectives**: *Changnienia amoena* is a rare and endangered terrestrial orchid endemic to China, valued for its ornamental and medicinal properties. However, limited genomic resources hinder its effective conservation strategies and sustainable utilization. This study aimed to generate comprehensive plastome resources and develop molecular markers to support the phylogenetics, identification, and conservation management of *C. amoena*. **Methods**: Genome skimming was employed to assemble and annotate the complete plastomes of seven geographically distinct *C. amoena* accessions. Comparative analyses were conducted to assess structural features and sequence divergence within *C. amoena* and across related species in the Calypsoinae subtribe. Phylogenetic relationships were inferred from protein-coding genes. Simple sequence repeats (SSRs), dispersed repeats, and hypervariable regions were identified from the plastomes, while nuclear SSRs were developed from assembled nuclear sequences. **Results**: All seven plastomes exhibited a conserved quadripartite structure with identical gene content and order, showing only minor variations in genome size. Sequence divergence was mainly confined to non-coding regions. Across *Calypsoinae* species, mycoheterotrophic taxa exhibited reduced plastomes. Phylogenetic analyses resolved four well-supported intergeneric clades within Calypsoinae and revealed a notable divergence between the HuNGZ accession and other *C. amoena* accessions, which otherwise showed low plastome-level differentiation. We also identified 69–74 plastome-derived SSRs, 22–25 dispersed repeats, and three hypervariable regions that may serve as informative molecular markers for *C. amoena*. Additionally, 16 polymorphic nuclear SSRs were developed from assembled nuclear sequences. **Conclusions**: These findings significantly expand the genomic resources available for *C. amoena* and provide essential insights for its phylogeny, molecular identification, conservation management, and future breeding efforts.

## 1. Introduction

The orchid family, Orchidaceae, is one of the most diverse and evolutionarily advanced groups of flowering plants, comprising over 30,000 species across more than 700 genera (www.catalogueoflife.org) [1]. Orchids account for nearly 10% of all angiosperms and 30% of monocotyledons [2,3,4]. Renowned for their striking flowers, distinctive growth forms, compact sizes, fragrant blooms, and foliar variegation, orchids hold significant economic value in horticulture and floriculture [5]. Beyond their ornamental value, orchids have long been used in traditional medicine for their bioactive compounds with antimicrobial, antioxidant, anti-inflammatory, and other therapeutic effects [6,7,8,9].

Moreover, orchids exhibit extraordinary ecological versatility, occurring from tropical rainforests to temperate woodlands, and are absent only from extreme deserts and polar regions [10,11]. Their widespread ecological success stems from a suite of specialized traits, including intricate floral morphologies that facilitate precise pollination, obligate mycorrhizal associations essential for seed germination and nutrient acquisition, and the evolution of Crassulacean Acid Metabolism (CAM), which enhances water-use efficiency in arid and epiphytic habitats [10,12,13]. Paradoxically, these adaptive innovations also contribute to their vulnerability. Their narrow habitat tolerances, dependence on specialized pollinators and fungi, and susceptibility to overharvesting have collectively contributed to their widespread endangerment [14,15]. Consequently, all orchid species are protected under the Convention on International Trade in Endangered Species of Wild Fauna and Flora (CITES; https://cites.org/), ensuring the strict regulation of international trade.

*Changnienia amoena* S.S. Chien, the sole species in the monotypic genus *Changnienia* within the subtribe Calypsoinae of the tribe Epidendreae, is an autotrophic, terrestrial orchid endemic to eastern and central China (https://duocet.ibiodiversity.net, accessed on 26 February 2025) [16]. This perennial herb typically inhabits nutrient-rich, moist, and partially shaded understory environments of mixed deciduous or evergreen forests at elevations of 400–1500 m [16,17]. Blooming from April to May, it produces solitary white or pink flowers marked with purplish-red spots, and is of high ornamental value [14,18]. Its pseudobulbs have traditionally been used to treat sores and snakebites [18]. Despite its great economic value, *C. amoena* populations have been severely fragmented and reduced due to anthropogenic disturbances such as agriculture, silviculture, urbanization, and the overcollection of its pseudobulbs [16,17,19]. In response, it was designated as Level II in China’s 2021 List of National Key Protected Wild Plants, and is currently listed as “Endangered” (EN) on the IUCN Red List [20]. Although its distribution, morphology, pollination biology, and ecological characteristics have been studied [17,19,21,22], genomic resources for this species remain extremely limited [23], hindering deeper genetic insights and the development of effective conservation strategies.

Recent advancements in next-generation sequencing (NGS) technologies have provided powerful tools to overcome the limitations posed by these scarce genomic resources. One particularly promising approach is genome skimming, a cost-effective, low-coverage sequencing method that targets high-copy genomic regions, including plastomes, ribosomal DNA, and simple sequence repeats (SSRs) [24,25]. This technique is especially well-suited for studies involving rare or endangered species like *C. amoena*, where conservation concerns often restrict the availability of DNA samples. Plastome sequences, in particular, have proven invaluable for phylogenetic and phylogeographic studies due to their unique characteristics, including a highly conserved structure, uniparental inheritance (usually maternal in angiosperms), low rates of nucleotide substitution, and an absence of recombination [26]. Recent studies have demonstrated the value of plastome phylogenomics in improving intraspecific phylogenetic resolution [27,28]. Moreover, comparative plastome analyses also enable the identification of variable regions, facilitating precise and efficient species discrimination [29]. In addition to plastome research, genome skimming also allows for the recovery of polymorphic nuclear SSRs (nSSRs), which serve as powerful molecular markers for evaluating genetic diversity, population structure, and gene flow [27,29]. Together, these genetic resources are essential for guiding targeted and effective conservation strategies.

In this study, we conducted genome skimming on seven geographically diverse *C. amoena* accessions to enrich their available genomic resources and advance our understanding of the plant’s intraspecific variation. Our objectives were as follows: (1) to analyze plastome variation among diverse *C. amoena* accessions and compare it with related Calypsoinae species to elucidate evolutionary patterns; (2) to reconstruct phylogenetic relationships both within *C. amoena* and among Calypsoinae species using plastome data; (3) to identify plastome-derived markers for *C. amoena*, including SSRs, dispersed repeats, and highly divergent regions; (4) to develop polymorphic nuclear SSR markers based on the assembled nuclear genomic sequences. These findings will provide essential resources for future research and offer valuable guidance for the conservation and sustainable utilization of this ecologically and economically important species.

## 2. Results and Discussion

### 2.1. Plastome Assembly and General Features

**Figure 1:**
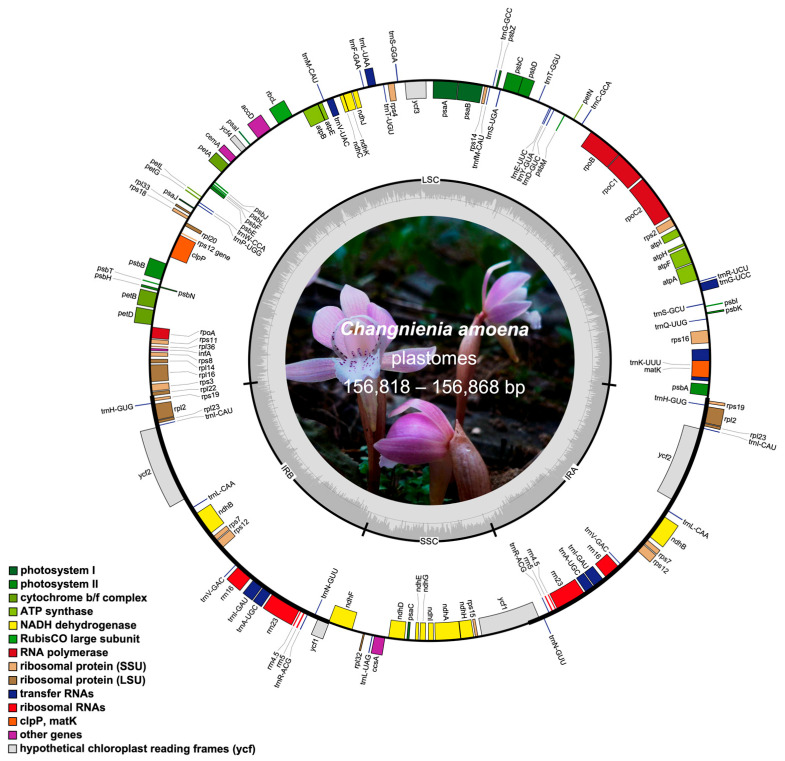
The circular map of the *C. amoena* plastomes. The genes located outside the circle are transcribed clockwise, while those inside are transcribed counterclockwise. Genes belonging to different functional categories are color coded. The inner gray ring indicates GC content (darker gray) and AT content (lighter gray). A photograph of *C. amoena* is shown within the inner circle.

The plastomes of all seven *C. amoena* accessions were successfully assembled into circular molecules, with lengths ranging from 156,818 bp to 156,868 bp and average embplant_pt base coverages of 171.4× to 429.3× (Figure 1, Table 1). Each plastome exhibited the typical quadripartite structure, a hallmark of most land plant chloroplast genomes, which consisted of four regions: a large single-copy (LSC) region (84,847 bp–84,883 bp), a small single-copy (SSC) region (18,136–18,161 bp), and two inverted repeat (IR) regions of 26,915 bp each. The GC contents of the whole plastome sequences (37.10%), as well as the LSC (34.80%), SSC (29.80%) and IR (43.20%) regions, were identical across the seven *C. amoena* accessions (Table 1). Each plastome encoded 113 unique genes, including 79 protein-coding genes (PCGs), 30 transfer RNA (tRNA) genes, and 4 ribosomal RNA (rRNA) genes, with 20 genes duplicated within the IR regions (Table 1). Among the 113 genes, 9 PCGs (*atpF*, *petB*, *petD*, *ndhB*, *ndhA*, *rpoC1*, *rpl16*, *rpl2*, *rps16*) and 6 tRNA genes (*trnK*-UUU, *trnG*-UCC, *trnL*-UAA, *trnV*-UAC, *trnI*-GAU, *trnA*-UGC) contained one intron, while 3 PCGs (*rps12*, *clpP*, and *ycf3*) contained two introns (Table S1). These findings are consistent with previous studies of other autotrophic species in the Calypsoinae subtribe, which also exhibit a conserved quadripartite plastome structure and similar genomic features [23,30].

### 2.2. Comparative Plastome Analyses Within and Between C. amoena and Closely Related Species

**Figure 2:**
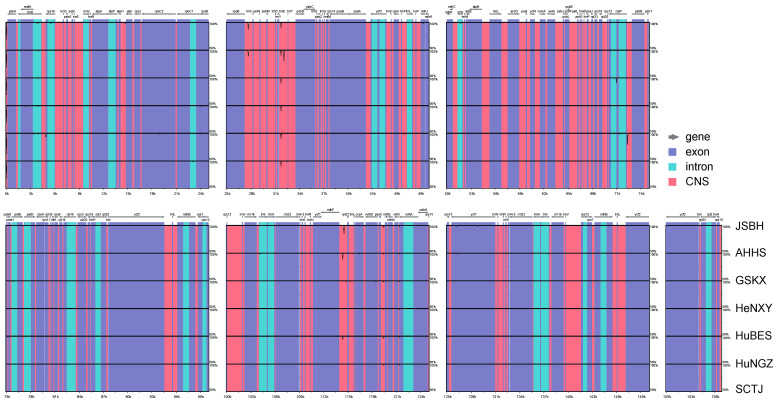
Sequence identity plots of the plastomes of the seven *C. amoena* accessions, with the accession JSBH used as the reference. Annotated genes are shown above the alignment, with gray arrows indicating gene orientation. The vertical axis represents percent identity (50–100%). The plastome regions are color-coded to distinguish exons, introns, and conserved non-coding sequences (CNSs).

Using the JSBH plastome as a reference, the seven newly sequenced *C. amoena* accessions were aligned and compared using mVISTA. The results revealed an exceptionally high level of sequence conservation (>99% identity) across the plastomes, with strong collinearity and overall structural homology. Only minor sequence divergence was observed in specific non-coding regions, particularly within intergenic spacers (Figure 2). This pattern was consistent with previous reports that protein-coding genes in land plant plastomes were generally highly conserved, whereas non-coding regions exhibited greater sequence variation [31,32].

**Figure 3:**
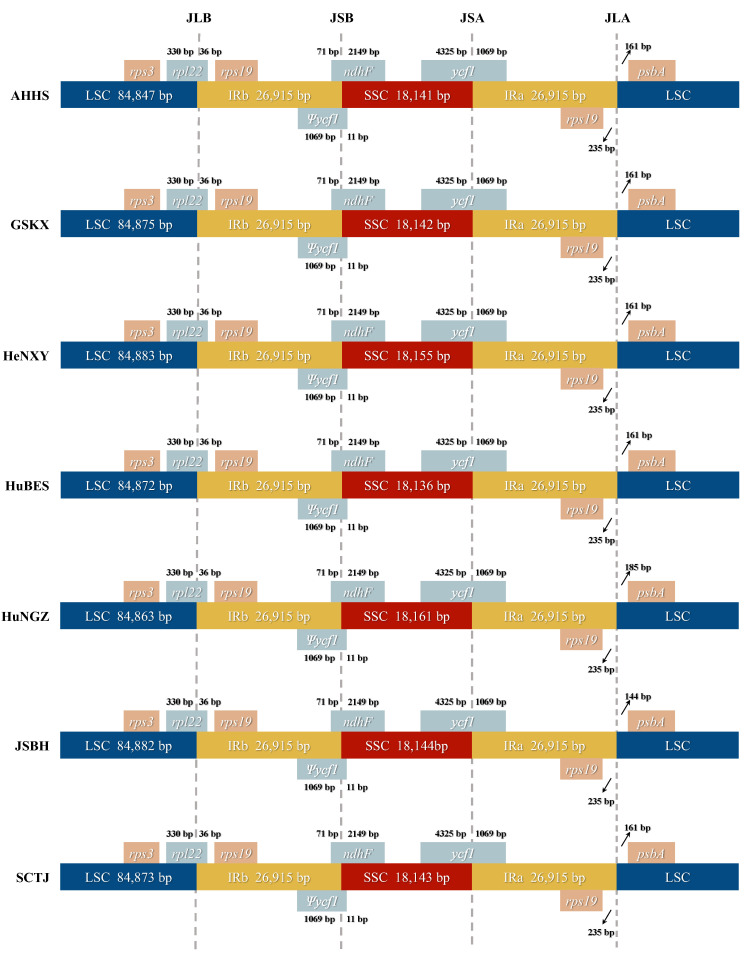
Comparison of IR/SC boundaries among seven *C. amoena* accessions.

All *C. amoena* plastomes also exhibited a high degree of conservation in the boundaries between IR and SC regions (Figure 3). The IRa region extended into the *ycf1* gene, resulting in a pseudogene fragment (^ψ^*ycf1*) of 1080 bp located at the IRb/SSC boundary (JSB). The *ndhF* gene overlapped with ^ψ^*ycf1* by 82 bp in all accessions. Additionally, the *rpl22* gene spanned the LSC/IRb region, with 36 bp located in the IRb region. The only notable variation among accessions was in the distance between the *psbA* gene and the IRa/LSC border: 144 bp in JSBH, 185 bp in HuNGZ, and 161 bp in the other five accessions (AHHS, GSKX, HeNXY, HuBES, and SCTJ) (Figure 3). The overall stability of the IR/SC junctions further highlights the conserved nature of plastome architecture in *C. amoena*, and the minor shifts in *psbA* positioning likely reflect neutral variation without functional consequence.

**Figure 4:**
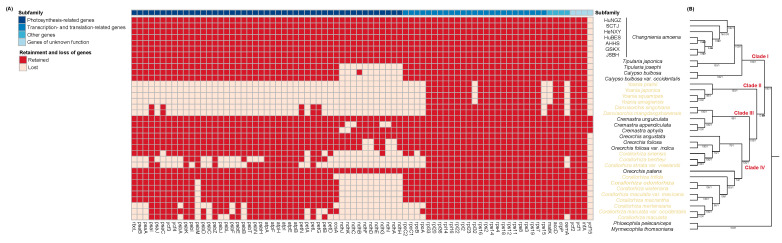
(**A**) Gene retention and loss among members of subtribe Calypsoinae. Red squares indicate retained genes, while beige squares indicate lost genes. Genes grouped by functional categories, including photosynthesis-related genes, transcription- and translation-related genes, other genes, and genes of unknown function, as indicated by color-coded bar at top. (**B**) Phylogenetic tree of Calypsoinae inferred using maximum likelihood (ML) and Bayesian inference (BI) methods. Leafless and mycoheterotrophic species highlighted in yellow. ML bootstrap values and BI posterior probabilities shown above branches (– indicates support values <50% or <0.5, respectively).

Further comparative analysis of plastome gene retention and loss within the subtribe Calypsoinae revealed stark contrasts between autotrophic and mycoheterotrophic taxa, largely reflecting their divergent life histories and nutritional strategies [33,34,35]. Autotrophic species, which synthesize their own organic compounds via photosynthesis, such as *C. amoena* and *Oreorchis patens*, retained complete quadripartite plastomes containing all 113 typical genes (79 protein-coding, 30 tRNA, 4 rRNA), including intact photosynthetic gene suites [23] (Figure 4A). In contrast, mycoheterotrophic species—plants that lack photosynthesis and instead obtain nutrients entirely through symbiotic fungi—like *Yoania* spp. exhibit highly reduced plastomes, with extensive gene loss, particularly of photosynthetic genes (Figure 4A). This degradation is primarily driven by the relaxed purifying selection on photosynthesis-related genes due to their loss of functional relevance. As these plants no longer perform photosynthesis and rely completely on fungal carbon sources, the plastid-encoded photosynthetic machinery becomes redundant and subject to degeneration [36]. These results further support the stepwise plastome degradation model proposed by Barrett & Davis (2012) [36], which suggests a stepwise loss beginning with photosynthetic genes, followed by genes related to transcription and translation, and eventually housekeeping genes.

Overall, *C. amoena* exhibited extremely low intraspecific plastome variation, while interspecific comparisons with closely related taxa revealed substantially greater divergence. This pattern suggests that plastome sequences, despite their conserved nature within species, retain strong discriminatory power at the species level. Although such low intraspecific variation is typical of plant species and may reflect slow plastome evolution or limited dispersal, it could also indicate reduced genetic diversity in *C. amoena* due to habitat fragmentation, population decline, or historical bottlenecks.

### 2.3. Phylogenetic Relationships Both Within C. amoena and Among Calypsoinae Species

Phylogenetic analyses using maximum likelihood (ML) and Bayesian inference (BI) revealed a distinct divergence between the HuNGZ accession and other *C. amoena* accessions (Figure 4B), with robust support values (bootstrap support [BS] = 100%, posterior probability [PP] = 1.0). In contrast, relationships among the remaining *C. amoena* accessions were poorly resolved, suggesting limited plastome-level differentiation despite their broad geographic distribution. This pattern contrasts sharply with nuclear RAPD results, which showed a clear population structure [14], highlighting cytonuclear discordance likely driven by differences in inheritance (maternally transmitted plastomes vs. biparentally inherited nuclear DNA), dispersal, and mutation rates. In *C. amoena*, seeds are minute and dust-like, typical of orchids, and are presumed to be dispersed over long distances by wind [37], facilitating the extensive homogenization of plastomes among populations. However, restricted pollen movement, driven by spatial isolation and low population densities, limits nuclear gene flow and contributes to stronger population structure in nuclear markers [14]. Moreover, the relatively slow mutation rate of plastomes likely contributes to the observed uniformity across plastome sequences.

At a broader scale, phylogenetic analysis of the Calypsoinae subtribe resolved four well-supported clades (Figure 4B). The basal Clade I (BS/PP = 100/1.0) consisted of *Changnienia* (represented by all seven accessions: AHHS, GSKX, HeNXY, HuBES, HuNGZ, JSBH, SCTJ), *Tipularia* (*T. japonica*, *T. josephi*), and *Calypso* (*C. bulbosa*, *C. bulbosa* var. *occidentalis*), with *Calypso* forming a sister group to the *Changnienia*–*Tipularia* lineage. This grouping is consistent with earlier morphological analyses, especially those focusing on floral traits and winter-leaf ecology, as well as with previous plastid-based phylogenies [38,39]. Clade II (BS/PP = 100/1.0) was formed solely by *Yoania* (*Y. prainii*, *Y. japonica*, *Y. squamipes*, *Y. amagiensis*), a monotypic genus known for its fully mycoheterotrophic lifestyle and distinct plastome characteristics, such as gene loss and rearrangements, consistent with the loss of photosynthetic function [40]. Although earlier phylogenies using *matK* and *ITS* sequences placed *Yoania* within the *Calypso* group [39,40], the exact placement and intrageneric relationships remained unresolved due to the limitations of short nuclear markers and the loss of key plastid genes [40]. Our plastome-based tree placed *Yoania* as sister to Clades III + IV, reinforcing its intermediate position within the subtribe (Figure 4B). However, the distinct plastome architecture of *Yoania* also raises taxonomic questions regarding its generic status and evolutionary independence [40]. These findings highlight the limitations of plastome data alone in fully mycoheterotrophic lineages and reinforce the importance of incorporating nuclear genomic evidence for robust phylogenetic resolution.

Clade III (BS/PP = 100/1.0) contained *Cremastra* (*C. unguiculata*, *C. appendiculata*, *C. aphylla*) and *Danxiaorchis* (*D. singchiana*, *D. mangdangshanensis*), the latter being a recently described genus. Clade IV (BS/PP = 74/1.0) consisted of the *Corallorhiza*–*Oreorchis* complex, but *Corallorhiza* was not monophyletic (Figure 4B). This unexpected pattern may result from historical hybridization, incomplete lineage sorting, or convergent evolution due to their shared mycoheterotrophic lifestyle [38]. Taken together, these results underscore the dynamic evolutionary history of Calypsoinae, where plastome data alone may be insufficient to fully resolve species relationships, especially in groups with suspected reticulate evolution. Future studies that incorporate nuclear genomic data and broader taxon sampling will be essential for resolving these phylogenetic relationships and elucidating the underlying evolutionary processes.

### 2.4. Plastome-Derived Marker Development for C. amoena

The identification of plastome-derived markers, such as simple sequence repeats (SSRs), dispersed repeats, and nucleotide diversity hotspots, is valuable for plant systematics, population genetics, and conservation biology [29,41,42]. Despite the growing availability of plastome sequences, previous studies often focused on interspecific comparisons, potentially underestimating intraspecific variation [27]. In this study, we analyzed whole plastome sequences from seven *C. amoena* accessions to identify SSRs, dispersed repeats, and highly variable regions for marker development.

**Figure 5:**
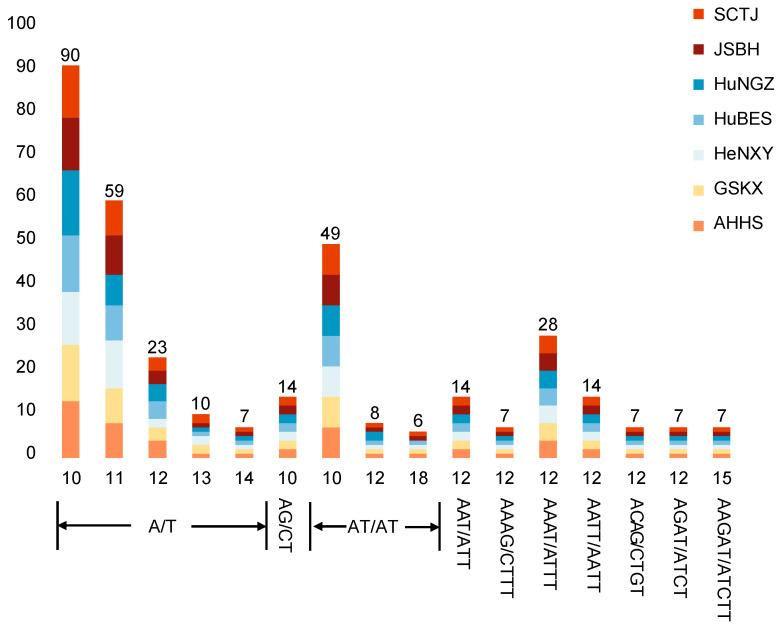
Distribution of plastome-derived SSRs in seven *C. amoena* accessions.

Plastome-derived SSRs have proven to be powerful molecular markers due to their uniparental inheritance, haploid and non-recombining nature, and high copy number, making them especially useful for tracking seed or pollen dispersal, identifying historical bottlenecks, and detecting founder events [43,44]. In this study, MISA analysis identified 350 plastome-derived SSRs across seven *C. amoena* accessions, with the number for each accession ranging from 49 (JSBH, SCTJ) to 51 (HuNGZ, HeNXY) (Figure 5, Table S2). These included 189 mononucleotide, 77 dinucleotide, 14 trinucleotide, 63 tetranucleotide, and 7 pentanucleotide repeats, respectively. Mononucleotide motifs were the most abundant (53.06–54.90%), and the SSRs were predominantly composed of A/T and AT/AT motifs (Figure 5, Table S2), reflecting the AT-rich nature of plastomes [45]. While SSR distribution was largely conserved, the HuNGZ accession showed slight differences, particularly in the number of (A/T)_10_, (AT/AT)_12_, and (AT/AT)_18_ SSRs (Figure 5, Table S2), consistent with its unique phylogenetic position (Figure 4B).

**Figure 6:**
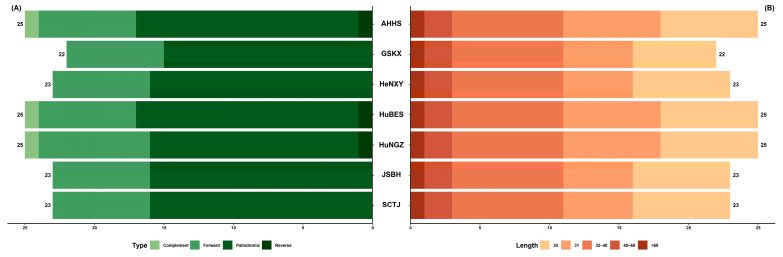
Summary statistics of repeat elements in seven *C. amoena* accessions. (**A**) Number of four types of dispersed repeats. (**B**) Number of different lengths of dispersed repeats.

Dispersed repeats, typically classified as forward, reverse, palindromic, and complementary, can mediate intragenomic instability and are often associated with dynamic regions of the plastome [46,47]. In this study, 22–25 dispersed repeats were detected across the *C. amoena* plastomes, with the majority (86.36–88.00%) ranging from 30 to 40 bp in length (Figure 6). Forward and palindromic repeats were predominant, accounting for 92.00–100.00% of all detected repeats. Complement and reverse repeats only occurred in the AHHS, HuBES, and HuNGZ accessions. Due to their sequence variability and potential roles in plastome evolution, these repeats provide valuable potential for marker development [46,48].

**Figure 7:**
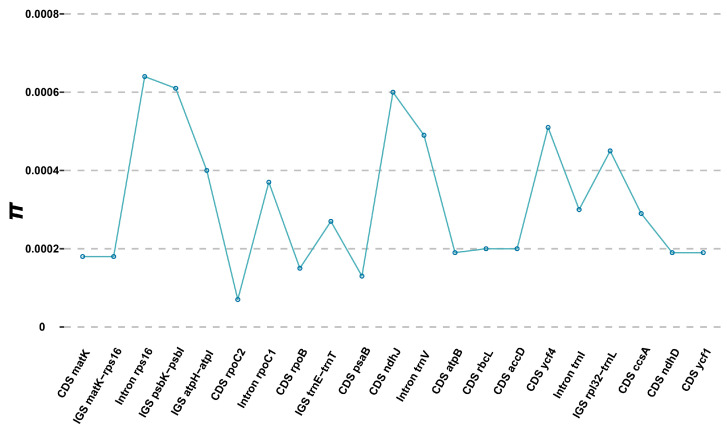
Nucleotide diversity (*π*) across 21 selected regions (12 CDS, 5 IGS, and 4 intron regions) extracted from alignment matrix of seven *C. amoena* accessions.

Plastomes were ideal resources for identifying divergent hotspots across various orchid lineages (e.g., [49,50]). Based on whole plastome alignments, we identified 21 regions exceeding 200 bp in length that contained mutations, including 12 coding sequences (CDS), 5 intergenic spacers (IGS), and 4 intron regions. The nucleotide diversity (*π*) for these regions ranged from 0.007% (CDS *rpoC2*) to 0.064% (Intron *rps16*) (Figure 7). The three most divergent regions, i.e., Intron *rps16*, IGS *psbK*-*psbI* and CDS *nhdJ*, with *π* > 0.060% exhibited greater divergence than commonly used DNA barcodes, such as CDS *matK*, CDS *rbcL* and IGS *trnH*-*psbA* [51], highlighting their potential for marker development in phylogenetic and phylogeographic studies.

### 2.5. Polymorphic Nuclear SSRs for C. amoena

Contrary to plastome-derived SSRs, nuclear SSRs (nSSRs) are biparentally inherited and typically exhibit higher polymorphism. These features make nSSRs a valuable complement to plastome-derived SSR analysis in plants, particularly for investigating genetic diversity, population structure, and adaptive variation [27,52]. In this study, 21 candidate polymorphic nuclear SSRs (PolynSSRs) were identified from nuclear genome scaffolds. Following the removal of low-quality loci exhibiting transferability (sequence similarity) <95% or a missing rate (MR) ≥50%, 18 high-quality candidate PolynSSRs were retained (Table S3). Primer pairs were successfully designed for 16 of these, representing 88.89% of the high-quality PolynSSRs. The final marker set included four trinucleotide repeats (25.0%), nine tetranucleotide repeats (56.25%), and three pentanucleotide repeats (18.75%) (Table S3).

## 3. Materials and Methods

### 3.1. Plant Materials, DNA Extraction, and Genome Sequencing

Field investigations and plant sampling were guided by species distribution data from the Flora of China, which reports *C. amoena* occurring in the Anhui, Hubei, Hunan, Jiangsu, Jiangxi, Shaanxi, Sichuan, and Zhejiang provinces. To capture a representative range of the species, seven accessions were sampled from five core provinces within its primary distribution (Anhui [AHHS], Hubei [HuBES], Hunan [HuNGZ], Jiangsu [JSBH], and Sichuan [SCTJ]), along with two adjacent regions (Gansu [GSKX] and Henan [HeNXY]) to account for potential peripheral variation (Table 1). Although the sample size was limited, these accessions spanned a broad geographic and ecological gradient across eastern and central China, representing key populations within the species’ natural range.

For each accession, healthy and fresh leaves were collected from a single individual and immediately dried with silica gel. To minimize any impact on this endangered species, only a small portion of leaf tissue was collected from each individual. Voucher specimens were deposited at the Herbarium of the Institute of Botany, Jiangsu Province, and the Chinese Academy of Sciences (NAS).

Genomic DNA was extracted from approximately 50 mg of silica-dried leaf tissue using the DNAsecure Plant Kit (Tiangen Biotech, Beijing, China) following the manufacturer’s protocol. DNA quality was assessed by 1% agarose gel electrophoresis, and DNA concentration was quantified using a Qubit 4.0 Fluorometer (Thermo Fisher Scientific, Waltham, MA, USA). Approximately 1 μg of high-quality DNA per sample was used to construct paired-end short-read sequencing libraries with an insert size of 350 bp for each sample using the MGIEasy universal DNA library prep kit (MGI, Shenzhen, China) according to the manufacturer’s guidelines. The libraries were quantified, pooled equimolarly, and sequenced on the DNBSEQ-T7 platform (BGI, Shenzhen, China) to generate approximately 10 Gb of raw data per sample. Trimmomatic v.0.36 [53] was used to trim sequencing adapters, reads with a quality score <20 over a sliding window size of 4 bp, and reads with a sequence length <50 bp.

### 3.2. Plastome Assembly and Annotation

High-quality clean reads were assembled into complete plastomes using GetOrganelle v.1.7.6 [54] with the following parameters: -R 15 -k 21,45,65,85,105 -F embplant_pt. Assembly graphs were visualized using Bandage v.0.8.1 [55] to confirm the circular structure of the plastomes. The initial annotation was conducted using the MAFFT v.7 plugin [56] within Geneious Prime^®^ 2022.0.1, aligning the newly assembled plastomes to the previously published plastome of *C. amoena* (MN047293) as a reference. The annotations were manually checked to verify the intron/exon boundaries and the accuracy of the start/stop codons. All newly generated plastome sequences were deposited in GenBank (accession numbers: PV612362-OQ526068). The final circular plastome maps were generated using OrganellarGenomeDRAW (OGDRAW) v.1.3.1 [57].

### 3.3. Whole Plastome Sequence Comparison

Whole plastome sequence comparisons among *C. amoena* accessions were conducted using the mVISTA program (http://genome.lbl.gov/vista/mvista/submit.shtml, accessed on 26 February 2025), employing the LAGAN alignment mode with default parameters [58]. The JSBH plastome sequence was designated as the reference, and alignments were visualized using the VISTA viewer [59]. To identify potential expansions or contractions in the inverted repeat (IR) regions of the *C. amoena* plastomes, the four junctions between the inverted repeat (IR) regions and the large single-copy (LSC)/small single-copy (SSC) regions were analyzed using the Repeat Finder plugin implemented in Geneious Prime^®^ 2022.0.1 (https://www.geneious.com/plugins/repeat-finder/, accessed on 26 February 2025).

Furthermore, to compare plastome gene retention and loss between *C. amoena* and its close relatives within Calypsoinae, as well as between autotrophic and mycoheterotrophic lineages, 31 previously published plastome sequences spanning seven genera and encompassing both nutritional modes were retrieved from the NCBI database (https://www.ncbi.nlm.nih.gov, accessed on 26 February 2025) for comparative analysis.

### 3.4. Phylogenetic Analyses

Phylogenetic relationships were inferred using both maximum likelihood (ML) and Bayesian inference (BI) approaches, based on the complete set of protein-coding genes from seven sampled *C. amoena* accessions and 28 previously published Calypsoinae plastomes, with *Phloeophila pelecaniceps* and *Myrmecophila thomsoniana* designated as outgroups. The protein-coding genes were aligned using MAFFT v.7 [56] and then concatenated into a single alignment matrix in Geneious Prime^®^ 2022.0.1. The best-fit nucleotide substitution model (GTR + I + G) was determined using jModelTest v.2.1.4 [60]. ML analyses were conducted using RAxML v.8.2.12 [61] on the CIPRES Science Gateway platform, with 1000 bootstrap replicates. BI analyses were performed using MrBayes v.3.2.7 [62] with two independent runs of 1 × 10^6^ generations. Each run consisted of four Markov Chain Monte Carlo (MCMC) chains (three heated and one cold), sampling every 1000 generations. The first 25% of sampled trees were discarded as burn-in, and a majority-rule consensus tree was then constructed to estimate posterior probabilities (PP).

### 3.5. Development of Plastome-Based Markers for C. amoena

Simple sequence repeats (SSRs) in the assembled *C. amoena* plastomes were identified using the MISA-web application [63] (https://webblast.ipk-gatersleben.de/misa/, accessed on 26 February 2025). The input FASTA files of the complete plastome sequences were uploaded to the platform, and the detection criteria were set as follows: mononucleotide repeats with a minimum of 10 repeat units, dinucleotide repeats with ≥5 units, trinucleotide repeats with ≥4 units, and tetra-, penta-, and hexanucleotide repeats with ≥3 units. Compound SSRs with a maximum interruption of 100 bp between two SSRs were also included.

Repetitive sequences, including forward (direct), reverse, complement, and palindromic repeats, were detected using the REPuter online program [64], with a minimum repeat length of 30 bp, a sequence identity of at least 90%, and a maximum Hamming distance of 3.

To pinpoint highly variable regions (“divergence hotspots”), the plastome sequences from all sampled *C. amoena* accessions were aligned using the MAFFT v.7 plugin [56] in Geneious Prime^®^ 2022.0.1. The alignment was manually inspected and partitioned into coding sequences (CDSs), introns, and intergenic spacer (IGS) regions based on the plastome annotation. Only regions longer than 200 bp and containing at least one polymorphic site were retained. Nucleotide diversity (*π*) for these regions was calculated using DnaSP v.6.12.03 [65], with default sliding window settings.

### 3.6. Polymorphic Nuclear SSRs Development

To develop polymorphic nuclear SSRs (PolynSSRs) for *C. amoena*, genome skimming data from these seven accessions were aligned to the reference genome of the closely related species *Ophrys sphegodes* (https://www.ncbi.nlm.nih.gov/datasets/genome/GCA_040285675.1, accessed on 26 February 2025) using BWA-MEM v.0.7.17 [66]. This step aimed to remove mitochondrial and plastome-derived reads. The resulting SAM files were sorted and converted to Binary Alignment/Map (BAM) format using SAMtools v.1.9 [67]. The filtered nuclear reads were then de novo assembled into scaffolds using SOAPdenovo v.1.0.4 [68], with scaffolds shorter than 500 bp discarded to ensure quality. PolynSSRs were identified from the assembled nuclear sequences using the CandiSSR pipeline [69]. Default parameters were applied, except for the flanking sequence length, which was increased to 200 bp to optimize primer design. Finally, primers for each identified PolynSSR locus were automatically designed using Primer3 [70].

## 4. Conclusions

In this study, we present the first comprehensive plastome analysis and genomic resource development for *C. amoena* using genome skimming data. We successfully assembled and characterized seven complete plastomes from geographically distinct accessions, all showing conserved gene content, structural organization, and IR/SC boundary regions. Comparative analyses revealed low overall plastome variation, with divergence primarily localized in intergenic spacers. Comparisons of gene content within the subtribe Calypsoinae further highlighted notable plastome reductions in mycoheterotrophic lineages. Phylogenetic analyses based on protein-coding genes resolved four well-supported intergeneric clades within Calypsoinae and revealed that, apart from the divergent HuNGZ accession, *C. amoena* accessions exhibit minimal plastome-level differentiation. A total of 350 plastome-derived SSRs and three divergent hotspots were identified, alongside the development of 16 high-quality polymorphic nuclear SSRs. These molecular markers provide valuable tools for future conservation genetic studies targeting population structure, gene flow, and adaptive variation. Overall, these findings underscore the utility of genome skimming for developing informative markers in rare species and lay a foundation for deeper investigations into the evolutionary and conservation dynamics of this endangered orchid.

## Figures and Tables

**Table 1:** Summary of characteristics of seven *C. amoena* plastomes.

Accession	AHHS	GSKX	HeNXY	HuBES	HuNGZ	JSBH	SCTJ
Locality	Huoshan, Anhui	Kangxian, Gansu	Xinyang, Henan	Enshi, Hubei	Guzhang, Hunan	Baohua, Jiangsu	Tongjiang, Sichuan
Plastome Size (bp)	156,854	156,868	156,856	156,847	156,838	156,818	156,846
Average base coverage (×)	429.3	351.2	317.8	244.9	171.4	363.2	399.2
LSC length (bp)	84,863	84,883	84,882	84,875	84,872	84,847	84,873
SSC length (bp)	18,161	18,155	18,144	18,142	18,136	18,141	18,143
IR length (bp)	26,915	26,915	26,915	26,915	26,915	26,915	26,915
GC content (%)	37.10%	37.10%	37.10%	37.10%	37.10%	37.10%	37.10%
LSC GC content (%)	34.80%	34.80%	34.80%	34.80%	34.80%	34.80%	34.80%
SSC GC content (%)	29.80%	29.80%	29.80%	29.80%	29.80%	29.80%	29.80%
IR GC content (%)	43.20%	43.20%	43.20%	43.20%	43.20%	43.20%	43.20%
Total genes	113	113	113	113	113	113	113
Total PCGs	87	87	87	87	87	87	87
Total tRNAs	38	38	38	38	38	38	38
Total rRNAs	8	8	8	8	8	8	8
Duplicated genes	20	20	20	20	20	20	20
GenBank accession number	PV612366	PV612364	PV612367	PV612363	PV612365	PV612362	PV612368

## Data Availability

The raw sequencing data of *C. amoena* have been deposited under NCBI BioProject PRJNA1264711 with accession nos. SRR33616569–SRR33616575.

## Supplementary Materials

The following supporting information can be downloaded at: https://www.mdpi.com/article/10.3390/genes16070723/s1, Table S1: Gene compositions in plastomes of *Changnienia amoena*; Table S2: Plastome-derived SSRs in seven *C. aamoena* plastomes; Table S3: Polymorphic nuclear SSRs across seven *C. amoena* accessions.

## References

[1] Wang Y.J., Wang H.C., Ye C., Wang Z.P., Ma C.B., Lin D.L., Jin X.H. (2024). Progress in systematics and biogeography of Orchidaceae. Plant Divers..

[2] Dressler R.L. (1993). Phylogeny and Classification of the Orchid Family.

[3] Hossain M.M., Kant R., Van P.T., Winarto B., Zeng S., Teixeira da Silva J.A. (2013). The application of biotechnology to orchids. Crit. Rev. Plant Sci..

[4] Christenhusz M.J., Byng J.W. (2016). The number of known plants species in the world and its annual increase. Phytotaxa.

[5] Hinsley A., De Boer H.J., Fay M.F., Gale S.W., Gardiner L.M., Gunasekara R.S., Kumar P., Masters S., Metusala D., Roberts D.L. (2018). A review of the trade in orchids and its implications for conservation. Bot. J. Linn. Soc..

[6] Choudhary D., Mashkey V.K., Goutam E., Shrivastava M., Rawat M., Kumari A., Tripathi V. (2023). Medicinal orchids: Traditional uses and recent advances. Ann. Phytomed..

[7] Lüning B., Withner C.L. (1974). Alkaloid content of Orchidaceae. The Orchids Scientific Studies.

[8] Kimura K. (1936). New species of *Dendrobium* from the Chinese drug Shih-hu. J. Shanghai Sci. Inst. Sect. III.

[9] Kaushik P. (1983). Ecological and Anatomical Marvels of the Himalayan Orchids.

[10] Zhang S.B., Yang Y.J., Li J.W., Qin J., Zhang W., Huang W., Hu H. (2018). Physiological diversity of orchids. Plant Divers..

[11] Chase M.W. (2005). Classification of Orchidaceae in the age of DNA data. Curtis’s Bot. Mag..

[12] Silvera K., Santiago L.S., Cushman J.C., Winter K. (2009). Crassulacean acid metabolism and epiphytism linked to adaptive radiations in the Orchidaceae. Plant Physiol..

[13] Givnish T.J., Spalink D., Ames M., Lyon S.P., Hunter S.J., Zuluaga A., Iles W.J., Clements M.A., Arroyo M.T., Leebens-Mack J. (2015). Orchid phylogenomics and multiple drivers of their extraordinary diversification. Proc. R. Soc. B.

[14] Li A., Ge S. (2006). Genetic variation and conservation of *Changnienia amoena*, an endangered orchid endemic to China. Plant Syst. Evol..

[15] Fay M.F., Chase M.W. (2009). Orchid biology: From Linnaeus via Darwin to the 21st century. Ann. Bot..

[16] Fu L., Chin C.M. (1992). China Plant Red Data Book.

[17] Xiong G.M., Xie Z.Q., Xiong X.G., Fan D.Y., Ge S. (2003). The biology and community characteristics of *Changnienia amoena* distributed in southern part of Shennongjia region. Acta Ecol. Sin..

[18] Jiang W.M., Yang G.M., Zhang C.L., Fu C.X. (2011). Species composition and molecular analysis of symbiotic fungi in roots of *Changnienia amoena* (Orchidaceae). Afr. J. Microbiol. Res..

[19] Sun H.Q., Luo Y.B., Alexandersson R., Ge S. (2006). Pollination biology of the deceptive orchid *Changnienia amoena*. Bot. J. Linn. Soc..

[20] Liu M.X., Wang X.W., Yang C.L. (2025). Exploring the potential distribution areas of *Changnienia amoena* and its pollinators in China based on MaxEnt and GTWR models. J. Nat. Conserv..

[21] Chen X.Q., Ji Z.H. (1998). The Orchids of China.

[22] Wang N.H., Lu Y., Cheng Z.L. (1994). Observation of biological properties and preliminary study on reproduction of *Changnienia amoena* Chien. Chin. Bull. Bot..

[23] Yi X.G., Li M.Z., Chen L., Wang X.R. (2020). The complete chloroplast genome of *Changnienia amoena* SS Chien (Orchidaceae) and its phylogenetic implication. Mitochondrial DNA Part B.

[24] Dodsworth S. (2015). Genome skimming for next-generation biodiversity analysis. Trends Plant Sci..

[25] Straub S.C., Parks M., Weitemier K., Fishbein M., Cronn R.C., Liston A. (2012). Navigating the tip of the genomic iceberg: Next-generation sequencing for plant systematics. Am. J. Bot..

[26] Birky Jr C.W., Maruyama T., Fuerst P. (1983). An approach to population and evolutionary genetic theory for genes in mitochondria and chloroplasts, and some results. Genetics.

[27] Hu K., Chen M., Li P., Sun X.Q., Lu R.S. (2023). Intraspecific phylogeny and genomic resources development for an important medical plant *Dioscorea nipponica*, based on low-coverage whole genome sequencing data. Front. Plant Sci..

[28] Lu R.S., Hu K., Sun X.Q., Chen M. (2024). Low-coverage whole genome sequencing of diverse *Dioscorea bulbifera* accessions for plastome resource development, polymorphic nuclear SSR identification, and phylogenetic analyses. Front. Plant Sci..

[29] Lu R.S., Yang T., Chen Y., Wang S.Y., Cai M.Q., Cameron K.M., Li P., Fu C.X. (2021). Comparative plastome genomics and phylogenetic analyses of Liliaceae. Bot. J. Linn. Soc..

[30] Chen X.Y., Xiang X.G., Liu X.D., Li W.Y., Wu X.C., Zhou Y.D., Yang B.Y., Luo H.L. (2023). Comparison of chloroplast genomes of *Calypsoinae* species (Orchidaceae) living on different lifeforms. Res. Sq..

[31] Shaw J., Lickey E.B., Schilling E.E., Small R.L. (2007). Comparison of whole chloroplast genome sequences to choose noncoding regions for phylogenetic studies in angiosperms: The tortoise and the hare III. Am. J. Bot..

[32] Lu R.S., Chen M., Feng Y., Yuan N., Zhang Y.M., Cao M.X., Liu J., Wang Y., Hang Y.Y., Sun X.Q. (2022). Comparative plastome analyses and genomic resource development in wild rice (*Zizania* spp., Poaceae) using genome skimming data. Ind. Crops Prod..

[33] Li Z.H., Jiang Y., Ma X., Li J.W., Yang J.B., Wu J.Y., Jin X.H. (2020). Plastid Genome Evolution in the Subtribe Calypsoinae (Epidendroideae, Orchidaceae). Genome Biol. Evol..

[34] Yang J.X., Peng S., Wang J.J., Ding S.X., Wang Y., Tian J., Yang H., Hu G.W., Wang Q.F. (2021). Morphological and genomic evidence for a new species of *Corallorhiza* (Orchidaceae: Epidendroideae) from SW China. Plant Divers..

[35] Lee S.Y., Meng K.K., Wang H.W., Zhou R.C., Liao W.B., Chen F., Zhang S.Z., Fan Q. (2020). Severe plastid genome size reduction in a mycoheterotrophic orchid, *Danxiaorchis singchiana*, reveals heavy gene loss and gene relocations. Plants.

[36] Barrett C.F., Davis J.I. (2012). The plastid genome of the mycoheterotrophic *Corallorhiza striata* (Orchidaceae) is in the relatively early stages of degradation. Am. J. Bot..

[37] Ferdy J.B., Loriot S., Sandmeier M., Lefranc M., Raquin C. (2001). Inbreeding depression in a rare deceptive orchid. Can. J. Bot..

[38] Barrett C.F., Freudenstein J.V., Skibicki S.V., Sinn B.T., Chung S.W., Hsu T.C., Liao W., Lee S.Y., Luo Y.B., Yukawa T. (2025). Phylogenomics and intergenomic conflict in a challenging orchid clade (Calypsoinae): Monophyly of *Corallorhiza*, paraphyly of *Oreorchis*, and resurrection of *Kitigorchis*. Bot. J. Linn. Soc..

[39] Freudenstein J.V., Yukawa T., Luo Y.B. (2017). A reanalysis of relationships among Calypsoinae (Orchidaceae: Epidendroideae): Floral and vegetative evolution and the placement of *Yoania*. Syst. Bot..

[40] Liu Z., Lee S.Y., Yeh C.L., Averyanov L.V., Liao W., Suetsugu K. (2024). Plastome analysis elucidates the phylogenetic placement of the mycoheterotrophic genus *Yoania* (Orchidaceae) and its plastomic degeneration during the evolution of mycoheterotrophy. Bot. J. Linn. Soc..

[41] Gu C.H., Tembrock L.R., Zheng S.Y., Wu Z.Q. (2018). The complete chloroplast genome of *Catha edulis*: A comparative analysis of genome features with related species. Int. J. Mol. Sci..

[42] Dong W.P., Liu J., Yu J., Wang L., Zhou S.L. (2012). Highly Variable Chloroplast Markers for Evaluating Plant Phylogeny at Low Taxonomic Levels and for DNA Barcoding. PLoS ONE.

[43] Provan P., Powell W., Hollingsworth P.M. (2001). Chloroplast microsatellites: New tools for studies in plant ecology and evolution. Trends Ecol. Evol..

[44] Zhou Q.Y., Cai H.X., Liu Z.H., Yuan L.X., Yang L., Yang T., Li B., Li P. (2022). Development of genomic resources for *Wenchengia alternifolia* (Lamiaceae) based on genome skimming data. Plant Divers..

[45] Kuang D.Y., Wu H., Wang Y.L., Gao L.M., Zhang S.Z., Lu L. (2011). Complete chloroplast genome sequence of *Magnolia kwangsiensis* (Magnoliaceae): Implication for DNA barcoding and population genetics. Genome.

[46] Li H.M., Wu M.S., Lai Q., Zhou W., Song C.F. (2023). Complete chloroplast of four *Sanicula* taxa (Apiaceae) endemic to China: Lights into genome structure, comparative analysis, and phylogenetic relationships. BMC Plant Biol..

[47] Zhou T., Zhu H.H., Wang J., Xu Y.C., Xu F.S., Wang X.M. (2020). Complete chloroplast genome sequence determination of *Rheum* species and comparative chloroplast genomics for the members of Rumiceae. Plant Cell Rep..

[48] Liang H., Zhang Y., Deng J.B., Gao G., Ding C.B., Zhang L., Yang R.W. (2020). The Complete Chloroplast Genome Sequences of 14 *Curcuma* Species: Insights into Genome Evolution and Phylogenetic Relationships Within Zingiberales. Front. Genet..

[49] Smidt E.d.C., Páez M.Z., Vieira L.d.N., Viruel J., de Baura V.A., Balsanelli E., de Souza E.M., Chase M.W. (2020). Characterization of sequence variability hotspots in Cranichideae plastomes (Orchidaceae, Orchidoideae). PLoS ONE.

[50] Kim Y.K., Jo S., Cheon S.H., Joo M.J., Hong J.R., Kwak M., Kim K.J. (2020). Plastome evolution and phylogeny of Orchidaceae, with 24 new sequences. Front. Plant Sci..

[51] Hollingsworth P.M., Li D.Z., van der Bank M., Twyford A.D. (2016). Telling plant species apart with DNA: From barcodes to genomes. Philos. Trans. R. Soc. B.

[52] Aecyo P., Marques A., Huettel B., Silva A., Esposito T., Ribeiro E., Leal I.R., Gagnon E., Souza G., Pedrosa-Harand A. (2021). Plastome evolution in the Caesalpinia group (Leguminosae) and its application in phylogenomics and populations genetics. Planta.

[53] Bolger A.M., Lohse M., Usadel B. (2014). Trimmomatic: A flexible trimmer for Illumina sequence data. Bioinformatics.

[54] Jin J.J., Yu W.B., Yang J.B., Song Y., DePamphilis C.W., Yi T.S., Li D.Z. (2020). GetOrganelle: A fast and versatile toolkit for accurate de novo assembly of organelle genomes. Genome Biol..

[55] Wick R.R., Schultz M.B., Zobel J., Holt K.E. (2015). Bandage: Interactive visualization of de novo genome assemblies. Bioinformatics.

[56] Katoh K., Standley D.M. (2013). MAFFT multiple sequence alignment software version 7: Improvements in performance and usability. Mol. Biol. Evol..

[57] Greiner S., Lehwark P., Bock R. (2019). OrganellarGenomeDRAW (OGDRAW) version 1.3.1: Expanded toolkit for the graphical visualization of organellar genomes. Nucleic Acids Res..

[58] Brudno M., Do C.B., Cooper G.M., Kim M.F., Davydov E., Green E.D., Sidow A., Batzoglou S., Program N.C.S. (2003). LAGAN and Multi-LAGAN: Efficient tools for large-scale multiple alignment of genomic DNA. Genome Res..

[59] Frazer K.A., Pachter L., Poliakov A., Rubin E.M., Dubchak I. (2004). VISTA: Computational tools for comparative genomics. Nucleic Acids Res..

[60] Darriba D., Taboada G.L., Doallo R., Posada D. (2012). jModelTest 2: More models, new heuristics and parallel computing. Nat. Methods.

[61] Stamatakis A. (2014). RAxML version 8: A tool for phylogenetic analysis and post-analysis of large phylogenies. Bioinformatics.

[62] Ronquist F., Teslenko M., Van Der Mark P., Ayres D.L., Darling A., Höhna S., Larget B., Liu L., Suchard M.A., Huelsenbeck J.P. (2012). MrBayes 3.2: Efficient Bayesian phylogenetic inference and model choice across a large model space. Syst. Biol..

[63] Beier S., Thiel T., Münch T., Scholz U., Mascher M. (2017). MISA-web: A web server for microsatellite prediction. Bioinformatics.

[64] Kurtz S., Phillippy A., Delcher A.L., Smoot M., Shumway M., Antonescu C., Salzberg S.L. (2004). Versatile and open software for comparing large genomes. Genome Biol..

[65] Rozas J., Ferrer-Mata A., Sánchez-DelBarrio J.C., Guirao-Rico S., Librado P., Ramos-Onsins S.E., Sánchez-Gracia A. (2017). DnaSP 6: DNA sequence polymorphism analysis of large data sets. Mol. Biol. Evol..

[66] Li H. (2013). Aligning sequence reads, clone sequences and assembly contigs with BWA-MEM. arXiv.

[67] Li H., Handsaker B., Wysoker A., Fennell T., Ruan J., Homer N., Marth G., Abecasis G., Durbin R. (2009). 1000 Genome Project Data Processing Subgroup. The sequence alignment/map format and SAMtools. Bioinformatics.

[68] Li R.Q., Zhu H.M., Ruan J., Qian W.B., Fang X.D., Shi Z.B., Li Y.R., Li S.T., Shan G., Kristiansen K. (2010). De novo assembly of human genomes with massively parallel short read sequencing. Genome Res..

[69] Xia E.H., Yao Q.Y., Zhang H.B., Jiang J.J., Zhang L.P., Gao L.Z. (2016). CandiSSR: An efficient pipeline used for identifying candidate polymorphic SSRs based on multiple assembled sequences. Front. Plant Sci..

[70] Koressaar T., Remm M. (2007). Enhancements and modifications of primer design program Primer3. Bioinformatics.

