# Supplementary material for: Genome Skimming Reveals Plastome Conservation, Phylogenetic Structure, and Novel Molecular Markers in Valuable Orchid Changnienia amoena
Source: Genes (Basel). 2025 Jun 20;16(7):723. doi: 10.3390/genes16070723 (PMC12296057; doi:10.3390/genes16070723)
Supplement: Supplementary file 1 [file genes-16-00723-s001.zip › genes-3697133-supplementary/Table S1.pdf]

Table S1 Gene compositions in plastomes of *Changnienia amoena*

Category of genes	Group of genes	Name of gene
RNA genes	Ribosomal RNAs	*rrn16* (×2), *rrn23* (×2), *rrn4.5* (×2), *rrn5* (×2)
	Transfer RNAs	*trnK*-UUU[a], *trnQ*-UUG, *trnS*-GCU, *trnG*-UCC[a], *trnR*-UCU, *trnC*-GCA, *trnD*-GUC, *trnY*-GUA, *trnE*-UUC, *trnT*-GGU, *trnS*-UGA, *trnG*-GCC, *trnfM*-CAU, *trnS*-GGA, *trnT*-UGU, *trnL*-UAA[a], *trnF*-GAA, *trnV*-UAC[a], *trnM*-CAU, *trnW*-CCA, *trnP*-UGG, *trnH*-GUG (×2), *trnI*-GAU (×2), *trnL*-CAA (×2), *trnV*-GAC (×2), *trnI*-GAU[a] (×2), *trnA*-UGC[a] (×2), *trnR*-ACG (×2), *trnN*-GUU (×2), *trnL*-UAG
Photosynthesis related genes	Rubisco	*rbcL*
	Photosystem I	*psaB*, *psaA*, *psaI*, *psaJ*, *psaC*
	Assembly/stability of photosystem I	*ycf3*[b], *ycf4*
	Photosystem II	*psbA*, *psbK*, *psbI*, *psbM*, *psbD*, *psbC*, *psbZ*, *psbJ*, *psbL*, *psbF*, *psbE*, *psbB*, *psbT*, *psbN*, *psbH*
	ATP synthase	*atpA*, *atpF*[a], *atpH*, *atpI*, *atpE*, *atpB,*
	Cytochrome b/f complex	*petN*, *petA*, *petL*, *petG*, *petB*[a], *petD*[a]
	Cytochrome c synthesis	*ccsA*
	NADH dehydrogenease	*ndhJ*, *ndhK*, *ndhC*, *ndhB*[a] (×2), *ndhF*, *ndhD*, *ndhE*, *ndhG*, *ndhI*, *ndhA*[a], *ndhH*
Transcription and translation related genes	Transcription	*rpoC2*, *rpoC1*[a], *rpoB*, *rpoA*
	Ribosomal proteins (large units)	*rpl33*, *rpl20*, *rpl36*, *rpl14*, *rpl16*[a], *rpl22*, *rpl2* [a] (×2), *rpl23* (×2), *rpl32*
	Ribosomal proteins (small units)	*rps16*[a], *rps2*, *rps14*, *rps4*, *rps18*, *rps12*[b] (×2), *rps11*, *rps8*, *rps3*, *rps19* (×2), *rps7* (×2), *rps15*
Other genes	RNA processing	*matK*
	Fatty acid synthesis	*accD*
	Caseinolytic protease proteolytic subunit	*clpP*[b]
	Carbon metabolism	*cemA*
Genes of unknown function	Conserved reading frame	*ycf2* (×2), *ycf1* (×2)

Pseudogenes	*InfA*

[a] indicates the genes containing a single intron; [b] indicates the genes containing two introns; (×2) indicates genes duplicated in the IR regions.
